# A New Species of *Pareas* (Squamata, Pareidae) from Guangxi Province, China †

**DOI:** 10.3390/ani13132233

**Published:** 2023-07-07

**Authors:** Yanan Gong, Jiaxiang Wu, Song Huang, Yuhao Xu, Diancheng Yang, Yongjin Liu, Shengming Liang, Pingshin Lee

**Affiliations:** 1 Anhui Provincial Key Laboratory of the Conservation and Exploitation of Biological Resources, College of Life Sciences, Anhui Normal University, Wuhu 241000, China; yan_an_gong@sinoophis.com (Y.G.); snakeboy@sinoophis.com (D.Y.); 2 Huangshan Noah Biodiversity Institute, Huangshan 245000, China; wjxwcl@hotmail.com (J.W.); yuhao_xu@sinoophis.com (Y.X.); lsm@sinoophis.com (S.L.); 3 School of Life Sciences, Anhui Agricultural University, Hefei 230036, China; 4 Nanning Municipal Public Security Bureau, Nanning 530012, China; liuyj@sinoophis.com

**Keywords:** snail-eating snake, *Pareas baiseensis* sp. nov., taxonomy, phylogeny, morphology

## Abstract

**Simple Summary:**

A new species of snail-eating snake in the genus *Pareas* is described, from the Youjiang District, Baise City, Guangxi Zhuang Autonomous Region, China, based on one male and three juvenile specimens. The maximum likelihood analyses based on *Cytochrome b* (Cyt b) and *NADH dehydrogenase subunit 4* (ND4) indicated the new taxon is different from its congeners. Morphologically, the new species can be diagnosed from the other species by a combination of seven characters. The recognition of the new species brings the number of described *Pareas* species to 30.

**Abstract:**

We described a new species of genus *Pareas* from Baise City, Guangxi Zhuang Autonomous Region, China, based on morphological and molecular evidence. *Pareas baiseensis* sp. nov. is distinguished from its congeners by the combination of (1) Yellowish-brown body colouration; (2) Frontal subhexagonal to diamond-shaped with its lateral sides converging posteriorly; (3) The anterior pair of chin shields is longer than it is broad; (4) Loreal not in contact with the eye, prefrontal in contact with the eye, two or three suboculars; (5) Rows of 15–15–15 dorsal scales, five rows of mid-dorsal scales keeled at the middle of the body, one vertebral scale row enlarged; (6) 187–191 ventrals, 89–97 subcaudals, all divided, cloacal plate single; (7) Two postocular stripes, the nuchal area forming a dark black four-pointed fork collar with the middle tines shorter than the outside tines. The genetic divergence (uncorrected *p*-distance) between the new species and other representatives of *Pareas* ranged from 13.9% to 24.4% for *Cytochrome b* (Cyt b) and 12.1% to 25.5% for *NADH dehydrogenase subunit 4* (ND4). Phylogenetic analyses of mitochondrial DNA gene data recovered the new species from being the sister taxon to (*P. boulengeri + P. chinensis*) from China.

## 1. Introduction

The family Pareidae Romer, 1956 (Squamata, Serpentes) encompasses four genera [1,2], respectively, namely *Aplopeltura* Duméril, 1853, *Asthenodipsas* Peters, 1864, *Pareas* Wagler, 1830, and *Xylophis* Beddome, 1878 [3,4]. The Asian snail-eating genus *Pareas* Wagler, 1830, is widely distributed through the Oriental zoogeographic region and is the most species-diverse genus in the Pareidae. It differs from other Pareidae genera by having 15 rows of dorsal scales at the midbody; all subcaudals divided; preocular and subocular scales present; supralabials usually not in contact with the eye; no anterior single inframaxillary, and three pairs of chin shields [4,5,6]. The morphology of snakes of the genus *Pareas* is highly conservative, and the morphological differences between the species are subtle and difficult to distinguish. Recent studies have demonstrated that the species diversity of the genus *Pareas* has been seriously underestimated [4,7,8,9]. Since 2015, based on integrative taxonomic approaches incorporating molecular analyses and morphological comparisons, twelve new species have been described, and seven species have been resurrected [4,6,8,9,10,11,12,13,14,15].

During our field research in the Youjiang District, Baise City, Guangxi Zhuang Autonomous Region, China, in November 2022, we collected four specimens of *Pareas* that differed from all members of the genus. Based on conclusive morphological and molecular evidence, we describe them as a new species.

## 2. Materials and Methods

### 2.1. Sampling

The four individuals of snail-eating snakes (one male, three juveniles) were collected from Daleng Township, Youjiang District, Baise City, Guangxi Zhuang Autonomous Region, China, in November 2022. The collected specimens were fixed in approximately 95% ethanol and subsequently transferred to 75% ethanol for permanent storage. Liver tissue samples were preserved separately in 95% ethanol. The specimens examined in the present study were preserved and deposited at Anhui Normal University Museum (ANU).

### 2.2. Morphological Examination

Referring to the following literature, Dowling 1951, Vogel 2015, Wang et al., 2020, and Poyarkov et al., 2022 [4,6,11,16], a total of 21 morphological characters in four specimens of the new species were examined (Table 1). Morphological measurements (all in mm) included: snout-vent length (SVL); tail length (TaL); total length (TL); relative tail length (TaL/TL); head length from snout tip to jaw angles (HL); maximal head width (HW); and eye diameter (ED). Meristic characteristics evaluated were the number of dorsal scale rows counted at one head length behind head (ASR), at mid-body (MSR), namely at SVL/2, and at one head length before vent (PSR); number of enlarged vertebral scale rows (VSE); number of keeled dorsal scale rows at midbody (KMD); number of ventral scales (VEN); number of subcaudal scales (SC); number of cloacal plates (CP); number of supralabials (SL); number of infralabials (IL); number of anterior temporals (At); number of posterior temporals (Pt); number of loreals (LOR); number of preoculars (Preoc); number of suboculars (SoO); and number of postoculars (PoO). We recorded the values for paired head characteristics on both sides of the head (in a left/right order). We measured body and tail lengths with a measuring tape (to the nearest of 1 mm); all other measurements were taken using an electronic slide caliper (to the nearest 0.1 mm).

### 2.3. Molecular Phylogeny

Total genomic DNA was extracted from an ethanol-preserved liver or muscle tissue using Tissue DNA Kits (Takara Biotechnology (Dalian) Co., Ltd., Dalian, China). We amplified the fragments of *cytochrome b* (cyt b; primer L14910, H16064; Queiroz et al., 2002) and *NADH dehydrogenase subunit 4* (ND4; primer ND4F, ND4LEUR; Salvi et al. 2013) mtDNA genes, using the Polymerase Chain Reaction (PCR) [17,18]. The PCR products were sequenced at Shanghai Map Biotech Co., Ltd. The raw sequences were stitched using SeqMan in the DNAstar software package [19]. In addition to sequences of 26 species of the genus *Pareas*, six outgroup taxa [3,4,11,19,20,21,22,23,24] were downloaded from GenBank (Details on taxonomy, localities, GenBank accession numbers in Table 1) and aligned with the newly generated sequences using the software MEGA X [25].

A maximum likelihood (ML) tree was reconstructed using RaxML v7.2.6 using the GTRGAMMA model with 1000 ultrafast bootstrap (BS) replicates [26,27]. We also calculated the pairwise distances (*p*-distances) among ingroup taxa using the neighbour-joining method in MEGA X [25,28].

The electronic version of this article in Portable Document Format (PDF) will represent a published work according to the International Commission on Zoological Nomenclature (ICZN), and hence the new names contained in the electronic version are effectively published under that Code from the electronic edition alone. This published work and the nomenclatural acts it contains have been registered in ZooBank, the online registration system for the ICZN. The ZooBank LSIDs (Life Science Identifiers) can be resolved, and the associated information can be viewed through any standard web browser by appending the LSID to the prefix http://zoobank.org/ (accessed on 8 June 2023). The LSID for this publication is urn:lsid:zoobank.org:pub:73634CF1-1ACA-4C07-B8A2-3929E2306558.

## 3. Results

### 3.1. Phylogenetic Relationship

**Figure 1:**
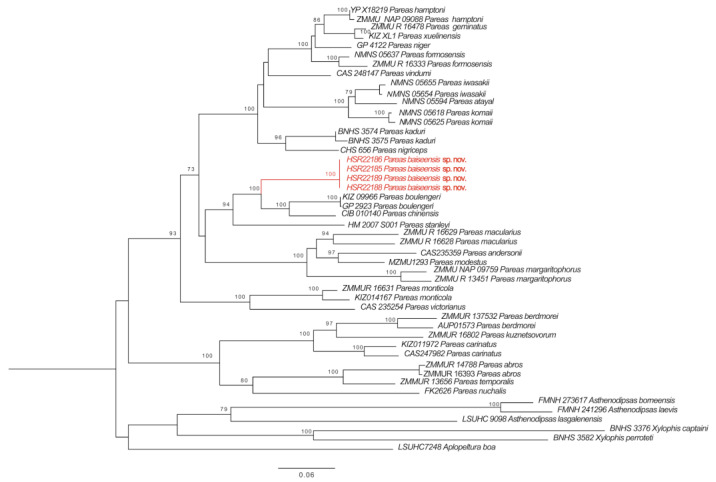
The maximum likelihood (ML) phylogenetic relationship trees based on concatenated Cyt b and ND4 fragments. Numbers near each node indicate the bootstrap support (The branch where the *P. baiseensis* sp. nov. is located is marked in red).

The newly generated sequences of Cyt b and ND4 genes of four specimens shared one haplotype for each gene. The sequences were submitted to GenBank (accession numbers, OQ054328 for Cyt b, OQ054329 for ND4). The *p*-distances based on fragments of Cyt b between the new species and other species of the genus *Pareas* varied from 13.9% (*Pareas boulengeri*) to 24.4% (*Pareas carinatus*) (Table 2), and those of ND4 varied from 12.1% (*Pareas boulengeri*) to 25.5% (*Pareas berdmorei*) (Table 3). Phylogenetic analyses of mitochondrial DNA data recovered the new species to be the sister taxon to (*P. boulengeri + P. chinensis*) from China (Figure 1). Combined with morphological data, the specimens from Baise, Guangxi, China, are considered to be a new species.

### 3.2. Taxonomic Account

*Pareas baiseensis* sp. nov. WU, GONG, HUANG, and XU

http://zoobank.org/984D3880-9849-46DB-A488-7D6542478AB7 (accessed on 8 June 2023)

**Figure 2:**
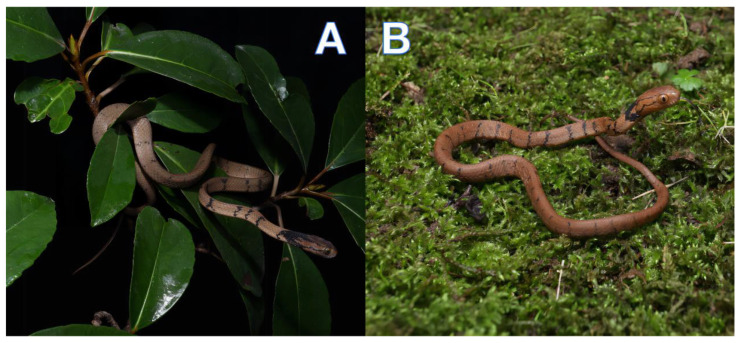
*Pareas baiseensis* sp. nov. in life. (**A**) Holotype male (ANU20220011); (**B**) Paratype juvenile (ANU20220014).

**Figure 3:**
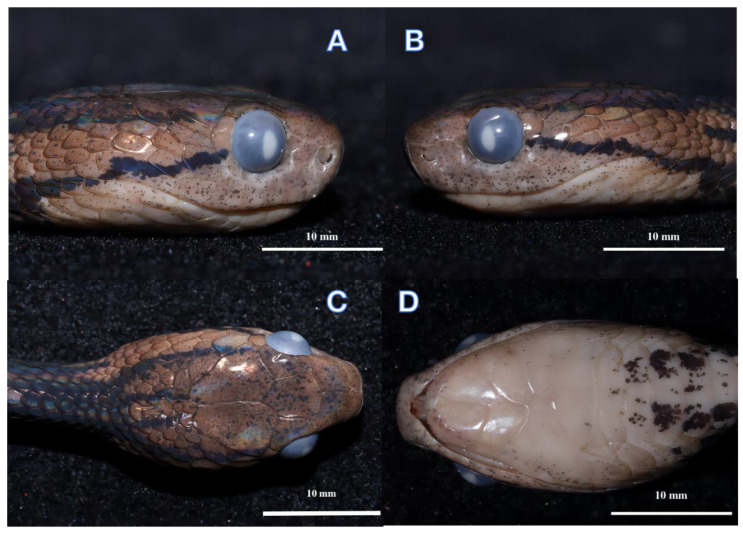
Holotype male (ANU20220011) of *Pareas baiseensis* sp. nov. in preservative. Right (**A**), Left (**B**), Ventral (**C**), Dorsal (**D**) views of the head.

**Figure 4:**
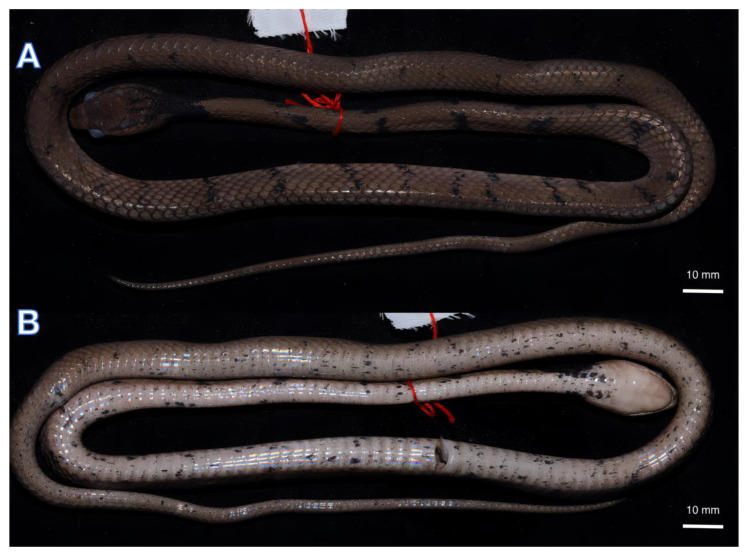
Dorsal (**A**) Vntral (**B**) views of the preserved holotype of *Pareas baiseensis* sp. nov. (ANU 20220011).

Figure 2, Figure 3 and Figure 4

Holotype. ANU20220011 (collection number HSR22185), an adult male (Figure 2A), was found in the Daleng Township, Youjiang District, Baise City, Guangxi Zhuang Autonomous Region, China (23.73521N, 106.39544E (DD); ca 781 m a.s.l.). The specimen was collected by Jiaxiang Wu and Yongjin Liu on 25 November 2022 and deposited at Anhui Normal University Museum.

Paratypes. ANU20220012 (collection number HSR22186), juvenile; ANU20220013 (collection number HSR22188, Figure 2B), juvenile; ANU20220014 (collection number HSR22189), juvenile, all with the same collecting information as the holotype.

### 3.3. Diagnosis

*Pareas baiseensis* sp. nov. is distinguished from all other *Pareas* by a combination of the following characteristics: (1) Yellow-brown body colouration; (2) Frontal subhexagonal to diamond-shaped with its lateral sides converging posteriorly; (3) The anterior pair of chin shields is longer than it is broad; (4) The loreal is not in contact with the eye, prefrontal in contact with the eye, two or three suboculars; (5) Rows of 15–15–15 dorsal scales, five rows of mid-dorsal scales keeled at the middle of the body, one vertebral scale row enlarged; (6) 187–191 ventrals, 89–97 subcaudals, all divided, cloacal plates single; (7) Two postocular stripes, the nuchal area forming a dark black four-pointed fork collar with the middle tines shorter than the outside tines.

### 3.4. Comparisons

*Pareas baiseensis* sp. nov. differs from *P. margaritophorus*, *P. macularius*, *P. modestus* and *P. andersonii* by having a light brown dorsum with irregular dark bands (vs. uniform grey to black to dark colouration, and with bicolored spots in *P. margaritophorus*, *P. macularius* and *P. andersonii*); 9/9 infralabials (vs. 7–8 infralabials); one vertebral scale row enlarged (vs. not enlarged); a higher number of ventrals (187–191 vs. 133–173); and a higher number of subcaudals (89–97 vs. 35–54). [4,6,7,8,28,29].

*Pareas baiseensis* sp. nov. differs from *P. nigriceps*, *P. niger*, and *P. stanleyi* by two or three suboculars (vs. one or suboculars fused with postoculars); the dorsal surface of the head is light brown with dark brown spots (vs a large black area on the back of the head); nuchal area forming a dark black four-pointed fork collar with the middle tines shorter than the outside tines. (vs. nuchal area no collar); 9/9 infralabials (vs. 7 or 8 infralabials); a higher number of ventrals (187–191 vs. 151–184); and a higher number of subcaudals (89–97 vs. 48–77) [4,6,7,9,29,30,31].

*Pareas baiseensis* sp. nov. differs from *P. abros*, *P. kuznetsovorum*, *P. temporalis*, *P. berdmorei*, *P. nuchalis* and *P. carinatus* by frontal subhexagonal with lateral sides converging posteriorly (vs. frontal hexagonal with lateral sides parallel to body axis); and the anterior pair of chin shields is longer than it is broad (vs. anterior pair of chin shields broader than long or slightly longer) [4,6,7,20,29,30,31,32].

*Pareas baiseensis* sp. nov. differs from *P. vindumi*, *P. victorianus*, and *P. monticola* by the loreal not contacting the eye (vs. the loreal contacting the eye); two or three suboculars (vs. one or suboculars fused with postoculars); five slightly keeled dorsal scale rows at midbody (vs. smooth or 7–11 keeled dorsal scale rows at midbody); and the nuchal area forming a dark black four-pointed fork collar with the middle tines shorter than the outside tines. (vs. nuchal area no collar) [6,7,10,29].

*Pareas baiseensis* sp. nov. differs from *P. hamptoni*, *P. kaduri*, *P. geminatus*, *P. xuelinensis*, *P. komaii*, *P. atayal*, *P. iwasakii*, and *P. formosensis* by nuchal area forming a dark black four-pointed fork collar with the middle tines shorter than the outside tines. (vs. nuchal area no collar); five slightly keeled dorsal scale rows at midbody (vs. smooth or 5–13 keeled dorsal scale rows at midbody); and two or three suboculars (vs. one or suboculars fused with postoculars) [6,7,9,10,11,12,29,30,31,32,33,34].

*Pareas baiseensis* sp. nov. differs from *Pareas dulongjiangensis* by the nuchal area forming a dark black four-pointed fork collar with the middle tines shorter than the outside tines (vs. two brownish-black longitudinal stripes running on each side of the neck leaving a pale central portion); the loreal not contacting the eye (vs. loreal contacting the eye); absence of preoculars (vs. preoculars being present); two or three suboculars (vs. suboculars fused with postoculars); a higher number of ventrals (187–191 vs. 182); and a higher number of subcaudals (89–97 vs. 76) [13].

*Pareas baiseensis* sp. nov. differs from *Pareas tigerinus* by the dorsal surface of the head, which is light brown with dark brown spots (vs. dorsal surface of head solid black or reddish-brown); two anterior temporals (vs. one anterior temporal); two or three suboculars (vs. suboculars fused with postoculars); a higher number of ventrals (187–191 vs. 160–171); and a higher number of subcaudals (89–97 vs. 62–64) [14].

*Pareas baiseensis* sp. nov. differs from *Pareas yunnanensis* by the dorsal surface of the head, which is light brown with dark brown spots (vs. dorsal surface of head is black); sides of the head with two lateral postorbital stripes (vs. no or one or two indistinct large black spots on each side of the head, no stripe on each side of the head); a higher number of infralabials (9 vs. 6–8); five slightly keeled dorsal scale rows at midbody (vs. 5–7 rows of middorsal scales keeled on the middle part of the body); a higher number of ventrals (187–191 vs. 169–175); and a higher number of subcaudals (89–97 vs. 59–65) [13].

*Pareas baiseensis* sp. nov. differs from *P. chinensis* by five slightly keeled dorsal scale rows at midbody (vs. smooth or seven keeled dorsal scale rows at midbody); one vertebral scale row enlarged (vs. three vertebral scale rows enlarged); two or three suboculars (vs. one subocular); nuchal area forming a dark black four-pointed fork collar with the middle tines shorter than the outside tines. (vs. nuchal area no collar); a higher number of ventrals (187–191 vs. 169–180); and a higher number of subcaudals (89–97 vs. 69–76) [4,6,7,9,29,35].

*Pareas baiseensis* sp. nov. differs from *P. boulengeri* by the loreal not contacting the eye (vs. the loreal contacting the eye); one vertebral scale row enlarged (vs. not enlarged); five slightly keeled dorsal scale rows at midbody (vs. smooth); two or three suboculars (vs. suboculars fused with postoculars); nuchal area forming a dark black four-pointed fork collar with the middle tines shorter than the outside tines. (vs. nuchal area no collar); a higher number of ventrals (187–191 vs. 164–187); and a higher number of subcaudals (89–97 vs. 63–78) [4,6,9,29,36].

### 3.5. Description of Holotype

An adult male, SVL 428 mm, TaL 151 mm, TL 579 mm, TaL/TL ratio 0.26; body slender, compressed; head elongate, clearly distinct from neck; snout round in dorsal view; eye slightly enlarged, pupil vertical and slightly elliptical; rostral slightly visible in dorsal view; frontal subhexagonal to diamond-shaped with its lateral sides converging posteriorly; nasal scale single; two prefrontals large, in contact with the eye; single loreal not in contact with the eye; temporals 2 + 3/3 + 3; 1/1 supraocular; 1/1 preocular; 4/4 suboculars; 1/1 postoculars; 8/8 supralabial scales; 9/9 infralabials; 191 (+1 preventral) ventrals; 15–15–15 dorsal scale rows, five rows of mid-dorsal scales keeled at the middle of the body; 97 subcaudals; cloacal plate single (Table 4).

Colouration: In life, the dorsal surface of the head is light brown with dark brown spots. The dorsum is brown with dark-brown speckling, and there are 34 irregular black cross-bands on the lateral sides of the body from neck to vent. The ventral is creamish–yellow with a few small black spots, the background colour gradually darkens to the rear, and the subcaudal scales are light brown. The sides of the head have two lateral postorbital stripes: the upper stripe extends from the temporal area backward extension to the dorsal scales of the neck, where it joins a large black collar around the nape, forming a dark black Ψ-shaped chevron pattern overall; the lower stripe extends backwards past the 9th supralabial, and at the throat contacting the four-pointed fork collar with the middle tines shorter than the outside tines. There are two black lines on the back of the parietal that extend back to the neck, with two lateral postorbital stripes; together, they form a dark black four-pointed fork collar.

In the preserved state, the colouration still resembles the specimen in life, but the dorsum colour fades to yellowish-brown (Figure 3 and Figure 4).

### 3.6. Variation

Measurements and scalation features of the type series (*n* = 4) are presented in Table 4. There is a certain variation observed in the number of ventrals, subcaudals, and temporals: ventrals (187–190, *n* = 4); subcaudals (89–96, *n* = 4); temporals (2 + 2, 2 + 3, 2 + 4, *n* = 4). Numerous irregular black cross-bands on the lateral sides of the body from neck to vent (32–40 bands, *n* = 4). The coloration features among the members of the type series were very similar.

### 3.7. Distribution

This species is currently only known from onelocality, Daleng Township, Youjiang District, Baise City, Guangxi Zhuang Autonomous Region, China. We found the snakes between 10:00 pm to 1:00 am after light rain in November 2022. The habitat environment was a well-preserved subtropical evergreen broad-leaved forest at elevations of 750–790 m.

## 4. Discussion

The phylogenetic results of Poyarkov et al. (2022) support the genus *Pareas sensu lato* being divided into two subgenera (*Pareas sensu stricto* and *Eberhardtia*) and six species groups; the subgenus *Pareas sensu stricto* includes two species groups (*P. carinatus* and *P. nuchalis* groups), the subgenus *Eberhardtia* includes four species groups (*P. chinensis*, *P. hamptoni*, *P. monticola* and *P. margaritophorus* groups). The members of the subgenus *Eberhardtia* differ from the members of the subgenus *Pareas* by the following combination of morphological characters: frontal subhexagonal to diamond-shaped with its lateral sides converging posteriorly; the anterior pair of chin shields is longer than it is broad; a single thin elongated subocular; and the ultrastructure of dorsal scales not ravine-like, having pore and arc structures, with arcs connecting to each other forming characteristic lines [4,8,37,38,39]. Our phylogenetic results support *Pareas baiseensis* sp. nov. belongs to the *P. chinensis* species groups in the subgenus *Eberhardtia*, but *Pareas baiseensis* sp. nov. have two or three suboculars, which is inconsistent with the diagnostic characteristic of subgenus *Eberhardtia.* Therefore, we propose deleting the diagnostic characteristic of a single thin elongated subocular.

The discovery of *Pareas baiseensis* sp. nov. increases the number of species of the *P.chinensis* species group to four species (*Pareas baiseensis* sp. nov., *P.boulengeri*, *P. stanleyi*, *P.chinensis*) in China. Among them, *P.boulengeri* is the most widely distributed, which is distributed in Guizhou, Sichuan, Yunnan, Chongqing, Henan, Hubei, Hunan, Guangxi, Guangdong, Jiangsu, Zhejiang, Anhui, Jiangxi, Fujian, Shaanxi, and Gansu. *P. stanleyi* is distributed in Fujian, Zhejiang, Jiangxi, Guizhou, Sichuan, Hunan and Guangxi; The distribution of *P.chinensis* in China is limited to the western and southern marginal mountains of the Sichuan Basin [6,7,14,15]; *Pareas baiseensis* sp. nov. is currently known only from the locality investigated, but Baise City is close to the borders of Yunnan and Vietnam, and this species may also occur in these adjacent areas.

The description of *Pareas baiseensis* sp. nov. from southern China brings the total number of recognized *Pareas* species to 30, of which 24 occur in China [2,4,14,15]. The genus *Pareas* has an ancient origin and poor migration ability, and the morphological difference between different species are subtle [9,21,40,41,42]; a large range of intensive sampling is helpful in discovering cryptic species, especially for some widely distributed types with unclear internal relationships, so sampling should be increased.

## 5. Conclusions

A new species of *Pareas*, *Pareas baiseensis* sp. nov., is described based on four specimens collected from the Youjiang District, Baise City, Guangxi Zhuang Autonomous Region, China, since Baise City is close to the borders of Yunnan and Vietnam and this species may also occur in these adjacent areas. However, their discovery is largely accidental, which makes it difficult for us to make accurate judgments on the distribution and population status of this new species. Further investigations will be necessary to assess the distribution and population status of this species.

## Figures and Tables

**Table 1:** Samples used for molecular phylogenetic analysis in this study.

No	Specimen ID	Species	Locality	cyt b	ND4
1	ANU000220008 = HSR22185	*Pareas baiseensis* sp. nov.	Baise, Guangxi, China	OQ054329	OQ054328
2	ANU000220009 = HSR22186	*Pareas baiseensis* sp. nov.	Baise, Guangxi, China	OQ054329	OQ054328
3	ANU000220010 = HSR22188	*Pareas baiseensis* sp. nov.	Baise, Guangxi, China	OQ054329	OQ054328
4	ANU000220011 = HSR22189	*Pareas baiseensis* sp. nov.	Baise, Guangxi, China	OQ054329	OQ054328
5	NMNS 05618	*P. komaii*	Taiwan, Taitung, Lijia	KJ642185	MW287056
6	NMNS 05625	*P. komaii*	Taiwan, Hualien	MZ712215	MZ712240
7	NMNS 05655	*P. iwasakii*	Japan, Okinawa, Ishigaki	KJ642160	—
8	NMNS 05654	*P. iwasakii*	Japan, Okinawa, Iriomote	MZ712216	—
9	NMNS 05594	*P. atayal*	Taiwan, Taoyuan, Beiheng	KJ642124	MW287041
10	CAS 235254	*P. victorianus*	Myanmar, Chin, Nat Ma Taung N.P.	MW438300	MW438302
11	KIZ 014167	*P. monticola*	China, Tibet (Xizang), Motuo	MK135109	MK805374
12	ZMMU R-16631	*P. monticola*	Myanmar, Sagaing, Ban Mauk	MW438296	MW438301
13	CAS235359	*P. andersonii*	Myanmar, Chin, Nat Ma Taung N.P.	MT968772	MW287040
14	ZMMU R-16628	*P. macularius*	Laos, Xaisomboun, Long Tien	MT968770	MZ712241
15	ZMMU R-16629	*P. macularius*	Myanmar, Sagaing, Ban Mauk	MT968771	MW287057
16	MZMU1293	*P. modestus*	India, Mizoram, Aizawl, Tanhril	MT968773	—
17	ZMMU R-13451	*P. margaritophorus*	Vietnam, Binh Puoc, Bu Gia Map N.P.	KJ642195	MW287058
18	ZMMU NAP-09759	*P. margaritophorus*	Thailand, Ratchaburi, Suan Phueng	MZ712217	MZ712243
19	KIZ 09966	*P. boulengeri*	China, Hubei, Jiannan	JF827678	JF827656
20	GP 2923	*P. boulengeri*	China, Guizhou, Jiangkou	MK135090	MK805355
21	CIB 010140	*P. chinensis*	China, Sichuan, Tianquan	JF827691	JF827669
22	HM 2007-S001	*P. stanleyi*	China, Guangxi, Guilin	JN230704	JN230705
23	CAS 248147	*P. vindumi*	Myanmar, Kachin, Lukpwi	MW287080	MW287059
24	CHS 656	*P. nigriceps*	China, Yunnan, Gaoligongshan N.R.	MK201455	—
25	BNHS 3575	*P. kaduri*	India, Arunachal Pradesh, Lohit, Kamlang W.S.	MT188734	—
26	BNHS 3574	*P. kaduri*	India, Arunachal Pradesh, Lohit, Kamlang W.S.	MW026190	—
27	ZMMU NAP-09088	*P. hamptoni*	Vietnam, Lao Cai, Bat Xat	MW287079	MW287053
28	YPX 18219	*P. hamptoni*	Myanmar, Kachin	MK135077	MK805342
29	ZMMU R-16478	*P. geminatus*	Thailand, Chiang Mai	MW287074	MW287050
30	KIZ-XL1	*P. xuelinensis*	China, Yunnan, Lancang, Xuelin	MW436709	—
31	NMNS 05637	*P. formosensis*	Taiwan, Nantou	MW287060	MW287042
32	ZMMU R-16333	*P. formosensis*	Vietnam, Gia Lai, Kon Chu Rang N.R.	MW287066	MW287048
33	GP 4122	*P. niger*	China, Yunnan, Kunming	MK135084	MK805349
34	AUP 01573	*P. berdmorei berdmorei*	Thailand, Chiang Mai	MZ712218	MZ712244
35	ZMMU R-13753-2	*P. berdmorei unicolor*	Vietnam, Dong Nai, Ma Da N.R.	MZ712224	MZ712250
36	ZMMU R-16802	*P. kuznetsovorum*	Vietnam, Phu Yen, Song Hinh	MZ712232	MZ712258
37	CAS 247982	*P. carinatus tenasserimicus*	Myanmar, Tanintharyi, Yaephyu	MZ712233	MZ712259
38	KIZ 011972	*P. carinatus*	Malaysia (peninsular)	MK135111	MK805376
39	ZMMU R-16393	*P. abros*	Vietnam, Quang Nam, Song Thanh N.P.	MZ712235	MZ712262
40	ZMMU R-14788	*P. abros*	Vietnam, Thua Thien-Hue, A Roang	MZ712237	MZ712264
41	ZMMU R-13656	*P. temporalis*	Vietnam, Lam Dong, Cat Loc	MZ712238	MZ712265
42	FK 2626	*P. nuchalis*	Brunei, Brunei Darussalam, Belait	MZ603794	U49311
43	LSUHC 7248	*Aplopeltura boa*	Malaysia, Sabah, Sepilok	KC916746	U49312
44	FMNH 241296	*Asthenodipsas laevis*	Malaysia, Penang, Pulau Pinang	KX660468	KX660596
45	LSUHC 9098	*Asthenodipsas lasgalenensis*	Malaysia, Pahang, Fraser’s Hill	KC916755	MZ712267
46	FMNH 273617	*Asthenodipsas borneensis*	Malaysia, Sarawak, Kelabit Highlands, Bario	KX660469	KX660597
47	BNHS 3376	*Xylophis captaini*	India	MK340914	MK340912
48	BNHS 3582	*Xylophis perroteti*	India	MN970042	MN970046

**Table 2:** Uncorrected *p*-distance based on a fragment of Cyt b among the genus *Pareas*.

	1	2	3	4	5	6	7	8	9	10	11	12	13	14	15
*1. P. baiseensis* sp. nov.	—														
*2. P. komaii*	0.194–0.200	0.017													
*3. P. iwasakii*	0.192–0.194	0.078–0.083	0.010												
*4. P. atayal*	0.205	0.085–0.093	0.069–0.072	—											
*5. P. victorianus*	0.189	0.187–0.192	0.194–0.198	0.194	—										
*6. P. monticola*	0.203–0.206	0.180–0.187	0.174–0.180	0.174–0.178	0.144–0.152	0.040									
*7. P. andersonii*	0.207	0.194–0.195	0.199–0.205	0.201	0.209	0.191–0.192	—								
*8. P. macularius*	0.187–0.194	0.181–0.194	0.191–0.197	0.189–0.193	0.192–0.193	0.173–0.176	0.135–0.145	0.114							
*9. P. modestus*	0.194	0.176–0.180	0.191–0.194	0.180	0.192	0.182–0.188	0.117	0.102–0.118	—						
*10. P. margaritophorus*	0.200–0.205	0.186–0.193	0.185–0.195	0.188–0.192	0.208–0.212	0.193–0.197	0.151–0.159	0.135–0.146	0.139–0.141	0.047					
*11. P. boulengeri*	0.139–0.141	0.178–0.181	0.173	0.181	0.193	0.185–0.188	0.198	0.183–0.186	0.191–0.193	0.190–0.192	0.002				
*12. P. chinensis*	0.146	0.181–0.185	0.177–0.181	0.187	0.176	0.182–0.186	0.192	0.176–0.179	0.188	0.187–0.192	0.089–0.090	—			
*13. P. stanleyi*	0.160	0.172–0.177	0.182–0.187	0.191	0.190	0.189–0.194	0.206	0.186–0.194	0.194	0.188–0.194	0.155	0.153	—		
*14. P. vindumi*	0.192	0.150	0.145–0.148	0.149	0.176	0.178–0.182	0.207	0.189–0.191	0.194	0.195–0.198	0.182	0.175	0.192	—	
*15. P. nigriceps*	0.169	0.162	0.159–0.161	0.157	0.191	0.188–0.191	0.188	0.183–0.193	0.164	0.178–0.19	0.169	0.162	0.190	0.123	—
*16. P. kaduri*	0.196–0.207	0.155–0.164	0.149–0.156	0.147–0.159	0.186–0.193	0.189–0.194	0.203–0.212	0.200–0.210	0.192–0.195	0.199–0.215	0.192–0.202	0.188–0.199	0.202–0.208	0.125–0.135	0.097–0.104
*17. P. hamptoni*	0.184–0.187	0.141–0.147	0.134–0.139	0.139	0.182–0.183	0.186–0.189	0.214	0.188–0.194	0.194	0.192–0.200	0.166–0.169	0.181	0.183–0.184	0.115–0.116	0.125–0.126
*18. P. geminatus*	0.200	0.152–0.153	0.135–0.139	0.142	0.192	0.194–0.198	0.219	0.192–0.199	0.206	0.199–0.202	0.169–0.170	0.187	0.193	0.122	0.132
*19. P. xuelinensis*	0.201	0.147	0.133–0.138	0.137	0.186	0.194–0.197	0.213	0.195–0.198	0.202	0.196–0.200	0.165–0.166	0.185	0.192	0.124	0.125
*20. P. formosensis*	0.189–0.195	0.147–0.154	0.133–0.148	0.145–0.149	0.175–0.184	0.188–0.200	0.212–0.214	0.193–0.201	0.198–0.203	0.192–0.196	0.165–0.173	0.171–0.181	0.193–0.195	0.115–0.120	0.125–0.133
*21. P. niger*	0.187	0.146–0.149	0.133–0.138	0.142	0.176	0.187–0.190	0.207	0.189–0.191	0.188	0.196–0.198	0.174–0.175	0.176	0.194	0.110	0.126
*22. P. berdmorei*	0.227–0.230	0.235–0.240	0.230–0.245	0.230–0.236	0.220–0.224	0.212–0.223	0.231–0.39	0.216–0.227	0.233–0.237	0.228–0.241	0.231–0.232	0.233–0.248	0.249–0.250	0.238–0.242	0.224–0.229
*23. P. kuznetsovorum*	0.235	0.239–0.241	0.236–0.241	0.229	0.226	0.221–0.225	0.233	0.216–0.222	0.238	0.227–0.232	0.220–0.222	0.230	0.249	0.231	0.239
*24. P. carinatus*	0.229–0.243	0.236–0.242	0.231–0.245	0.229–0.236	0.226–0.228	0.223–0.227	0.225–0.237	0.216–0.228	0.237–0.245	0.226–0.242	0.216–0.221	0.227–0.231	0.243–0.244	0.238–0.243	0.229–0.231
*25. P. abros*	0.243–0.244	0.228–0.232	0.225–0.233	0.226–0.227	0.242–0.244	0.227–0.229	0.234–0.235	0.246–0.242	0.231–0.232	0.241–0.250	0.233–0.234	0.238	0.255–0.257	0.244–0.245	0.232–0.236
*26. P. temporalis*	0.239	0.240	0.227–0.234	0.234	0.246	0.218–0.231	0.233	0.231–0.233	0.231	0.242–0.248	0.224–0.225	0.219	0.242	0.251	0.241
	**16**	**17**	**18**	**19**	**20**	**21**	**22**	**23**	**24**	**25**	**26**	**27**			
*1. P. baiseensis* sp. nov.															
*2. P. komaii*															
*3. P. iwasakii*															
*4. P. atayal*															
*5. P. victorianus*															
*6. P. monticola*															
*7. P. andersonii*															
*8. P. macularius*															
*9. P. modestus*															
*10. P. margaritophorus*															
*11. P. boulengeri*															
*12. P. chinensis*															
*13. P. stanleyi*															
*14. P. vindumi*															
*15. P. nigriceps*															
*16. P. kaduri*	0.015														
*17. P. hamptoni*	0.125–0.136	0.005													
*18. P. geminatus*	0.139–0.151	0.083–0.084	—												
*19. P. xuelinensis*	0.133–0.141	0.078–0.082	0.027	—											
*20. P. formosensis*	0.128–0.141	0.072–0.074	0.087–0.096	0.080–0.088	0.041										
*21. P. niger*	0.122–0.132	0.056–0.060	0.076	0.073	0.071–0.079	—									
*22. P. berdmorei*	0.242–0.251	0.227–0.246	0.245–0.255	0.244–0.251	0.241–0.248	0.230–0.236	0.071								
*23. P. kuznetsovorum*	0.228–0.233	0.230–0.231	0.247	0.243	0.230–0.232	0.223	0.121–0.125	—							
*24. P. carinatus*	0.221–0.244	0.231–0.232	0.237–0.242	0.237–0.241	0.233–0.240	0.229–0.233	0.130–0.149	0.128–0.133	0.080						
*25. P. abros*	0.248–0.257	0.231–0.236	0.231–0.232	0.230–0.231	0.231–0.236	0.226–0.227	0.208–0.218	0.206–0.209	0.216–0.230	0.015					
*26. P. temporalis*	0.249–0.252	0.237–0.238	0.248	0.246	0.234–0.240	0.236	0.203–0.209	0.196	0.194–0.197	0.127–0.132	—				
*27. P. nuchalis*	0.253–0.26	0.250	0.264	0.260	0.240–0.248	0.254	0.210	0.203	0.206–0.2	0.212–0.21	0.201	—			

**Table 3:** Uncorrected *p*-distance based on a fragment of ND4 among the genus *Pareas*.

	1	2	3	4	5	6	7	8	9	10	11	12	13	14	15	16	17	18	19	20	21	22
*1. P. baseensis* sp. nov	*—*																					
*2. P. komaii*	0.208	—																				
*3. P. ata* *yal*	0.202	0.076	—																			
*4. P. victorianus*	0.184	0.189	0.183	—																		
*5. P. monticola*	0.186–0.193	0.205–0.211	0.189–0.198	0.127–0.139	0.056																	
*6. P. andersonii*	0.180	0.195	0.189	0.183	0.186–0.194	—																
*7. P. macularius*	0.186–0.189	0.201–0.207	0.195–0.196	0.196–0.204	0.195–0.215	0.120	0.103															
*8. P. margaritophorus*	0.189	0.201–0.210	0.196–0.201	0.189–0.201	0.193–0.213	0.139–0.147	0.147–0.153	0.071														
*9. P. boulengeri*	0.121	0.190	0.184	0.180	0.178–0.181	0.179	0.181–0.196	0.177–0.187	—													
*10. P. chinensis*	0.122	0.192	0.021	0.184	0.174–0.184	0.182	0.193–0.201	0.190–0.208	0.101	—												
*11. P. stanleyi*	0.162	0.186	0.195	0.194	0.192–0.200	0.179	0.179–0.183	0.195–0.204	0.148	0.166	—											
*12. P. vindumi*	0.148	0.142	0.139	0.154	0.174–0.175	0.161	0.178	0.172–0.184	0.156	0.157	0.165	—										
*13. P. hamptoi*	0.174–0.177	0.172–0.175	0.163	0.169–0.175	0.177–0.186	0.189	0.196–0.204	0.189–0.201	0.178–0.181	0.165	0.204	0.128–0.131	0.006									
*14. P. geminatus*	0.178	0.172	0.160	0.172	0.183–0.192	0.179	0.186–0.207	0.199–0.208	0.181	0.181	0.197	0.134	0.053–0.056	—								
*15. P. formosensis*	0.171	0.157–0.174	0.156–0.171	0.174–0.183	0.190–0.205	0.188–0.198	0.199–0.211	0.195–0.201	0.189–0.199	0.174–0.183	0.189–0.194	0.123–0.139	0.076–0.100	0.082–0.107	0.051							
*16. P. niger*	0.180	0.162	0.160	0.184	0.189–0.198	0.188	0.195–0.204	0.184–0186	0.177	0.171	0.191	0.136	0.089–0.091	0.101	0.082–0.101	—						
*17. P. berdmorei*	0.242–0.255	0.224	0.211–0.221	0.211–0.230	0.204–0.216	0.203–0.212	0.225–0.234	0.213–0.227	0.215–0.227	0.224–0.228	0.236–0.239	0.201–0.213	0.205–0.215	0.207–0.218	0.208–0.222	0.222–0.230	0.066					
*18. P. kuznetsovorum*	0.228	0.213	0.211	0.215	0.195–0.202	0.214	0.216–0.237	0.202–0.219	0.199	0.218	0.224	0.193	0.189	0.198	0.187–0.219	0.213	0.140–0.142	—				
*19. P. carinatus*	0.224–0.231	0.211–0.216	0.210–0.211	0.205–0.207	0.204–0.218	0.229–0.230	0.230–0.242	0.211–0.218	0.210–0.221	0.227–0.228	0.224–0.225	0.198–0.204	0.196–0.210	02101–0.211	0.198–0.227	0.199–0.213	0.137–0.150	0.144–0.151	0.053			
*20. P. abros*	0.218–0.221	0.219–0.222	0.224	0.202	0.205–0.219	0.202–0.203	0.221–0.224	0.205–0.216	0.211–0.215	0.211–0.215	0.216–0.218	0.193	0.193–0.196	0.184	0.186–0.207	0.199–0.202	0.177–0.199	0.201	0.189–0.201	0.003		
*21. P. temporalis*	0.208	0.196	0.199	0.181	0.186–0.196	0.198	0.218	0.195–0.208	0.202	0.187	0.201	0.187	0.195	0.187	0.193–0.201	0.181	0.186–0.195	0.190	0.189–0.192	0.095–0.098	—	
*22. P. nuchalis*	0.228	0.236	0.233	0.234	0.208–0.210	0.197	0.221–0.233	0.211–0.234	0.222	0.224	0.228	0.199	0.207–0.210	0.202	0.198–0.215	0.211	0.178–0.181	0.195	0.187–0.189	0.172–0.175	0.172	—

**Table 4:** Measurements (mm) and pholidosis for the holotype and paratypes of *Pareas baiseensis* sp. nov.; for abbreviations, see the Section 2.

Voucher Number	ANU2022008 (Holotype)	ANU2022009 (Paratype)	ANU2022010 (Paratype)	ANU2022011 (Paratype)
collection number	HSR22185	HSR22186	HSR22188	HSR22189
SEX	male	juvenile	juvenile	juvenile
SVL	428	174	171	185
TaL	151	56	51	67
TL	579	230	222	252
TaL/TL	0.26	0.24	0.23	0.27
HL	19.6	11.1	10.9	12.3
HW	10.6	6.2	6.0	6.5
ED	4.1	2.6	2.6	2.5
ASR	15	15	15	15
MSR	15	15	15	15
PSR	15	15	15	15
VSE	1	1	1	1
KMD	5	5	5	5
VEN	191	190	187	189
SC	97	91	89	96
CP	entire	entire	entire	entire
SL	8/8	8/8	8/8	8/8
IL	9/9	9/9	9/9	9/9
At	2/3	2/2	2/2	2/2
Pt	3/3	3/3	3/4	3/2
LOR	1/1	1/1	1/1	1/1
Preoc	2/2	2/2	2/2	2/2
SoO	3/3	2/2	2/2	2/2
PoO	1/1	1/1	1/1	1/1

## Data Availability

The data presented in this study are available on request from the corresponding author.
